# Voriconazole-induced photocarcinogenesis is promoted by aryl hydrocarbon receptor-dependent COX-2 upregulation

**DOI:** 10.1038/s41598-018-23439-7

**Published:** 2018-03-22

**Authors:** Shigeki Ikeya, Jun-ichi Sakabe, Takahiro Yamada, Takafumi Naito, Yoshiki Tokura

**Affiliations:** 1 Department of Dermatology, Hamamatsu University School of Medicine, 1-20-1 Handayama, Higashi-ku, Hamamatsu, 431-3192 Japan; 2 Department of Hospital Pharmacy, Hamamatsu University School of Medicine, 1-20-1 Handayama, Higashi-ku, Hamamatsu, 431-3192 Japan

## Abstract

Voriconazole (VRCZ) induces the development of UV-associated skin cancers. The mechanism underlying the VRCZ-induced carcinogenesis has been largely unknown. Here, we showed that VRCZ metabolites plus UVA generated reactive oxygen species and resultant DNA damage of the epidermis, but did not induce substantial apoptosis in human keratinocytes (KCs). Furthermore, VRCZ *per se* stimulates aryl hydrocarbon receptor (AhR) and upregulates COX-2, which is a pivotal enzyme for the promotion of UV-associated tumors, in an AhR-ARNT dependent manner of the classical (genomic) pathway. Our findings suggest that the phototoxic moieties of VRCZ metabolites may participate in the initiation phase of VRCZ skin cancer, while VRCZ *per se* promotes the tumor development. Therefore, during VRCZ therapy, sun exposure protection is essential to prevent photocarcinogenesis caused by VRCZ metabolites plus UV. Chemoprevention with selective COX-2 inhibitors may be helpful to repress the development of skin cancers derived from DNA-damaged KCs.

## Introduction

Voriconazole (VRCZ) is a broad-spectrum triazole antifungal agent and is therapeutically effective for invasive aspergillosis and candidiasis. It is commonly administered to lung and hematopoietic cell transplant patients both prophylactically and as treatment for invasive fungal infection. This drug with high efficacy and availability has been used since its FDA approval in 2002^1–3^.

Although VRCZ is generally well tolerated^1^, there are serious side effects of vision change, hepatic enzyme abnormalities and photosensitivity^3^. Photosensitivity has been reported in 8–10% of patients receiving VRCZ^3–5^. Meanwhile, multifocal cutaneous squamous cell carcinoma (cSCC) secondary to prolonged VRCZ therapy was first described in 2007^4^. Cases of such an extraordinary harmful cutaneous adverse event of VRCZ, including cSCC and actinic keratosis (AK) have been accumulated^5–9^, strongly suggesting the VRCZ carcinogenicity. A recent case-control^10^ and retrospective cohort study^11^ also indicated that VRCZ is an independent risk factor for development of cSCC in lung transplant patients. Furthermore, even in non-transplant patients, if they were immunocompromised, VRCZ treatment induced multifocal and aggressive skin cancers^12,13^, further supporting the peculiar adverse effect of VRCZ on skin tumor development. Given that VRCZ-induced skin cancers arise only on the sun-exposed skin areas, the cutaneous carcinogenesis of VRCZ seems to be closely related to its photosensitivity^5^. However, there have been scarce studies on the mechanism of VRCZ carcinogenesis in relation to its photosensitive moiety.

In this study, we sought to investigate the UV-associated, tumor-initiating and -promoting actions of VRCZ. First, we used VRCZ metabolites. Although VRCZ does not absorb light in the UV spectrum, its primary hepatic metabolite, VRCZ *N*-oxide (VNO), has absorption spectra in UV^2^. Based on this unique characteristic, it is tempting to speculate that VRCZ metabolites are engaged in VRCZ photosensitivity^14^ and early carcinogenesis. Next, we focused on aryl hydrocarbon receptor (AhR), because another azole agent has an affinity to AhR^15^, and examined the ability of VRCZ to upregulate COX-2 in an AhR-dependent manner. Since COX-2 is critical enzyme for UV-induced skin cancer promotion, this signaling pathway possibly leads to skin tumor promotion as in breast cancer cells^16^. Results suggest that the phototoxic moieties of VRCZ metabolites may participate in the initiation phase of VRCZ skin cancer, while VRCZ *per se* promotes the tumor development.

## Results

### VRCZ metabolites plus UV generate ROS without substantial apoptosis: possible association with the initiation phase of carcinogenesis

**Figure 1:**
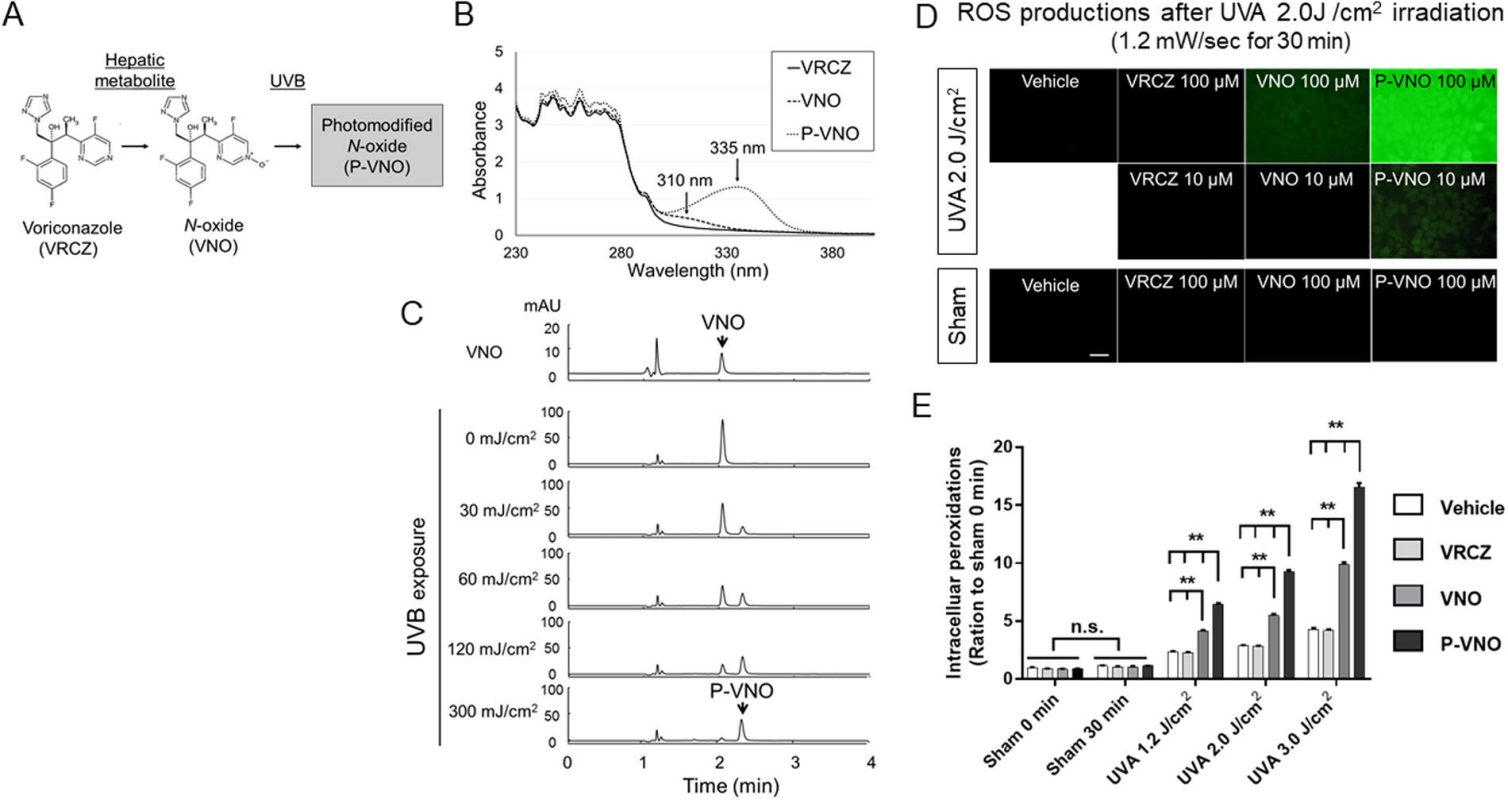
Characteristics of VRCZ and its metabolites (**A–C**). (**A**) VRCZ is converted to hepatic metabolite VNO, which is further converted to photo-modified-VNO (P-VNO) by UVB irradiation. The chemical structure of P-VNO is unknown. (**B**) The absorption spectra of VRCZ, N-oxide and P-N-oxide. P-N-oxide was generated by 500 mJ/cm^2^ UVB irradiation. Concentrations of the chemicals were 100 µM. Absorption spectra were recorded under the following settings-scan range 230 to 400 nm; slit width, 1.0 nm; scan rate of 5 nm/s. (**C**) HPLC chromatograms of VNO irradiated with varying doses of UVB. VNO solutions were exposed to 0-300 mJ/cm^2^ UVB at 305 nm, concentrated at 100 µM, and injected into the HPLC system. Retention times are 2.05 and 2.32 min for VNO and P-VNO, respectively. The wavelength of the photodiode array UV detector was set at 340 nm. (**D**) P-VNO generates significant amounts of ROS following 2.0 J/cm^2^ (1.2 mW/sec for 30 min) UVA irradiation in HaCaT KCs. The magnitude of ROS formation was evaluated with DCFH-DA (Green fluorescence). Immediately after irradiation, the cells were subjected to experimental treatments. (**E**) ROS was produced by P-VNO, depending on the dose of UVA (1.2 J/cm^2^; 18 min, 2.0 J/cm^2^; 30 min, 3.0 J/cm^2^; 45 min by 1.2 mW/sec). VNO plus UVA also yielded ROS to a lesser degree. “Sham 0 min” represents a no-treatment group, and “Sham 30 min” remained left for 30 min without UVA irradiation. Data are presented as means ± SEM (*n* = 4). Scale bar: 50 µm. Turkey’s all-pairwise-comparison test was performed. **P < 0.01.

VRCZ induces photosensitivity, but the action spectrum of the photosensitivity is uncertain. Although VRCZ is stable against UV, the main hepatic metabolite of the *N*-oxide form (VNO) with *N-*oxidation at the fluoropyrimidine ring has the maximum absorption spectrum within the UVB range^17^. By virtue of this absorption ability, VNO can be converted to photo-modified VNO (P-VNO) upon UVB exposure (Fig. 1A). In confirmation of the previous report^14^, we observed that the absorption spectrum of P-VNO is shifted to UVA range (peak at 335 nm, Fig. 1B). We also irradiated VNO with varying doses of UVB (0-300 mJ/cm^2^, peak at 305 nm) and subjected the irradiated substances to HPLC analysis, which demonstrated that VNO and P-VNO were eluted at 2.05 and 2.32 min, respectively (Fig. 1C). Thus, VNO was photo-modified and converted to P-VNO in an UVB dose-dependent manner.

To evaluate the ROS-producing ability of P-VNO upon UVA exposure, HaCaT KCs were incubated with DMSO as control, VRCZ, VNO and P-VNO, and irradiated with UVA at 2.0 J (1.2 mW/sec for 30 min)/cm^2^. Immediately after irradiation, the cells were subjected to experimental treatments. The ROS levels were assessed by using DCFH-DA. We found that P-VNO induced ROS production strongly at 100 µM and moderately at 10 µM after 2.0 J/cm^2^ UVA compared with VRCZ and VNO (Fig. 1D). The intracellular peroxidation was monitored in HaCaT cells treated with each chemical plus UVA at 0, 1.2, or 3.0 J (1.2 mW/sec for 0, 18, or 42 min)/cm^2^. Again, P-VNO produced ROS in an UVA dose dependent manner (Fig. 1E). VNO plus UVA also yielded ROS to a lesser degree.

**Figure 2:**
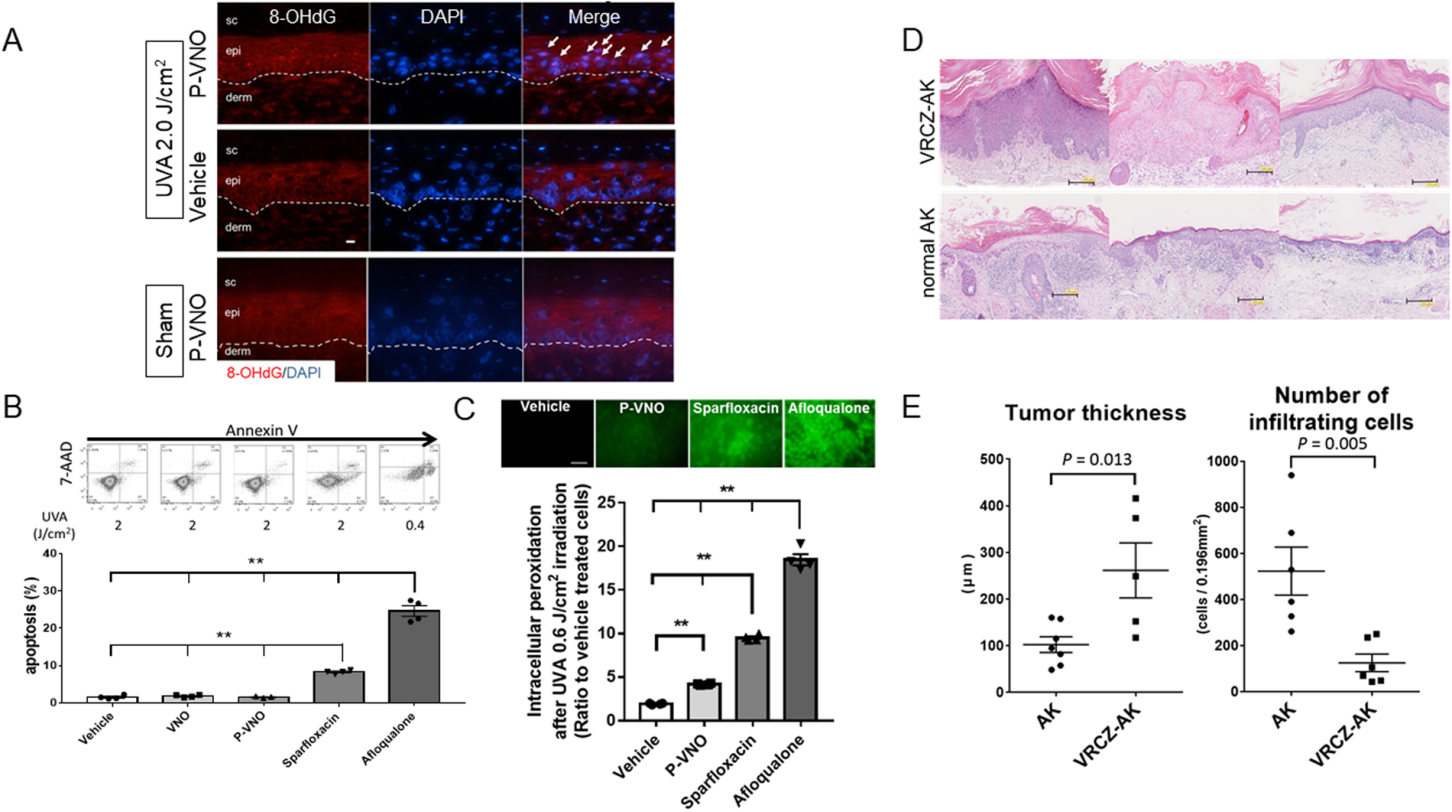
ROS-producing but low apoptosis-inducible potential of VRCZ. (**A**) Immunohistochemical staining for 8-hydroxy-2′-deoxyguanosine (8-OHdG; red) of human living skin equivalent (LSE) model incubated with P-VNO (top) or vehicle (DMSO; bottom) for 2 hours and irradiated with 2.0 J/cm^2^ (1.2 mW/sec for 30 min) UVA. Nuclei of KCs treated with P-VNO plus UVA were prominently stained for 8-OHdG. Arrows indicate 8-OHdG positive nuclei. DAPI, nuclear staining; sc, stratum corneum; epi, epidermis; derm, dermis. Dotted lines mark the dermoepidermal junction. Scale bar: 20 µm. (**B**) Representative dot-charts of Annexin-V/7-AAD bivariate flow cytometry of vehicle, VNO, P-VNO, sparfloxacin and afloqualone (from left to right). HaCaT KCs were pretreated with each chemical (100 µM) or vehicle and irradiated with the indicated dose of UVA. The lower left quadrant (Annexin-V^−^/7-AAD^−^) contains viable cells. The lower right quadrant (Annexin-V^+^/7-AAD^−^) represents apoptotic cells. The results were expressed as the mean percentage ± SEM of apoptotic cells (*n* = 4). Turkey’s all-pairwise-comparison test was performed. **P < 0.01. Panels A, C are representative images from 3 replicate experiments. (**C**) ROS productions by P-VNO, sparfloxacin and afloqualone (from left to right). HaCaT KCs were treatment with each chemicals (100 µM) for 30 minutes exposed to P-VNO. Then, they were irradiated with 0.7 J/cm^2^ (1.2 mW/sec for 10 min) UVA. Immediately after irradiation, KCs were subjected to ROS experiments. Panels is representative image from 2 replicate experiments and data are presented as means ± SEM (*n* = 4). Scale bar: 50 µm. (**D**) Histolopathology of AK from the patients treated with VRCZ (VRCZ-AK; top) and those without history of VRCZ therapy (non-VRCZ-AK, normal AK; bottom). VRCZ-AK shows epidermal hyperplasia and scarce infiltration of inflammatory cells compared with non-VRCZ-AK (H&E staining, original magnification 5×). Scale bar: 200 µm. (**E**) The tumor thickness is significantly higher and the number of infiltrating lymphocytes is significantly lower in the VRCZ-AK group than in the control AK groups. Bars indicate mean ± SEM (*n* = 5–7). Student’s *t* test.

P-VNO plus UVA elicits oxidative DNA damage of cultured monolayer KCs^14^. We further examined the DNA damage by P-VNO plus UVA in human living skin equivalent (LSE) model. LSE was incubated with P-VNO for 2 hours and irradiated with 2.0 J (1.2 mW/sec for 30 min)/cm^2^ UVA. DNA damage was evaluated by IHS for 8-OHdG (red) one hour after irradiation. Nuclei of KCs were prominently stained for 8-OHdG (Fig. 2A), a pre-mutagenic oxidation product of guanine in DNA^18^, suggesting the role of P-VNO plus UVA in photo-carcinogenesis of VRCZ.

Apoptosis is inducible by oxidative stress. We examined the effect of P-VNO plus UVA on HaCaT KCs apoptosis. KCs were incubated with or without VNO or P-VNO and exposed to UVA at 2.0 J (1.2 mW/sec for 30 min)/cm^2^. Sparfloxacin (antimicrobial fluoroquinolone)^19^ and afloqualone (muscle relaxant, a quinazolinone derivative)^20^ were used as positive controls. Apoptosis was assessed by flow cytometry at 8 hours after treatment. In the dot-plot histogram, the lower right quadrant represents early apoptotic cells that are positive only for Annexin V (Fig. 2B). We found that no substantial apoptosis was induced by VNO or P-VNO with UVA, while representative phototoxic chemicals, sparfloxacin and afloqualone (even with 0.4 J (1.2 mW/sec for 6 min)/cm^2^ UVA), produced high frequencies of apoptotic KCs. In addition, no apparent apoptosis of KCs was found in the treatment with P-VNO plus UVA at different time points (24 and 48 hours after UVA irradiation) (Supplemental Fig. 1). We compared the amounts of ROS production by other photosensitizers. The strong photosensitizers generated significantly higher amounts of ROS than did P-VNO (Fig. 2C). Thus, although P-VNO plus UVA affects KCs with oxidative stress, it seems that they are not efficiently eliminated by apoptosis because of the low ROS production by VRCZ metabolite plus UVA.

### Highly acanthotic and scarcely inflammatory histolopathology of VRCZ-induced AK

The above findings suggested that the metabolic and photo-induced products of VRCZ can participate in the VRCZ-induced carcinogenesis by means of the phototoxic moieties. Since VRCZ-induced cSCCs are highly extensive and aggressive than ordinary cSCCs^5,9,12^, it is considered that other factors underlie the mechanism. Therefore, we compared VRCZ-induced AKs with ordinary AKs to obtain the useful information on the mechanisms of VRCZ-induced skin tumors (*n* = 5–7, 2010–2016 in our university hospital). As represented by three cases from each group, VRCZ-AKs showed epidermal hyperplasia and scarce infiltration of inflammatory cells (Fig. 2D, top panel) compared with non-VRCZ-AKs (Fig. 2D, bottom panel). The tumor thickness was significantly higher and the number of infiltrating lymphocytes was significantly lower in the VRCZ-AK group than those in the control AK group (Fig. 2E). These findings provide an implication that VRCZ can strongly evoke the tumor formation of KCs under a certain immunocompromised condition and its tumorigenesis is not simply caused by photosensitivity.

### VRCZ upregulates AhR-mediated COX-2 expression and prostaglandin E_2_ (PGE_2_) production

**Figure 3:**
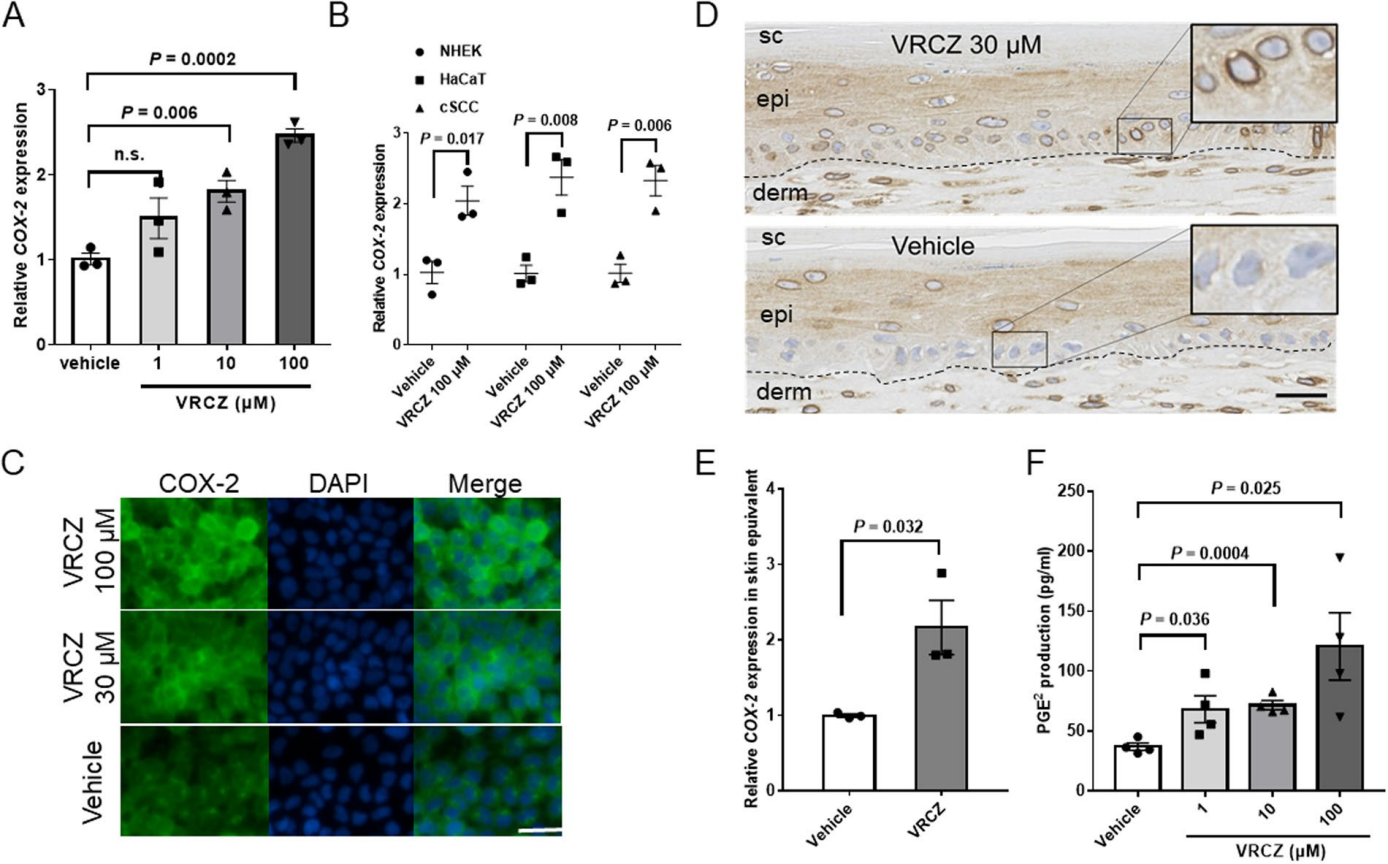
Up-regulation of COX-2 expression and PGE_2_ production by VRCZ. (**A**) After 2 hours incubation with VRCZ, *COX-2* mRNA expression relative to that of the vehicle-treated cells were examined in HaCaT KCs. Date are normalized to GAPDH and expressed as fold increase from 0.07% DMSO-treated control. Bars indicate mean ± SEM (*n* = 3). (**B**) After 2 hours incubation with VRCZ, *COX-2* mRNA expressions were examined in NHEKs, HaCaT KCs and cSCC cell lines. Bars indicate mean ± SEM (*n* = 3). (**C**) COX-2 staining on HaCaT cells incubated with 30 or 100 μM VRCZ or vehicle (0.07% DMSO) for 6 hours. Cells were fixed and stained with the polyclonal IgG rabbit anti-human COX-2 antibody followed by incubation with FITC-labeled anti-rabbit IgG secondary Ab. Scale bar: 20 µm. (**D**) LSEs were cultivated with or without VRCZ (30 μM) in the culture medium after airlift and harvested 2 weeks later. The paraffin sections were immunohistochemically stained with COX-2 antibody. Inset: prominent perinuclear staining in the basal and lower spinous layers of VRCZ-treated LSE. sc, stratum corneum; epi, epidermis; derm, dermis. Dotted lines mark the dermoepidermal junction. Scale bar: 50 μm. (**E**) *COX-2* mRNA levels in LSE after VRCZ treatment. Bars indicate mean ± SEM (*n* = 3). (**F**) HaCaT cells were treated with 1, 10 or 100 μM VRCZ, or vehicle (0.07% DMSO) for 6 hours. The PGE_2_ concentration in the supernatants was measured by ELISA. Bars indicate mean ± SEM (*n* = 4). Student’s *t* test. Panels C, D are representative images from 3 replicate experiments.

According to the results of UV and clinical examination, we next focus on the action of VRCZ *per se* in KCs. Especially, we examined whether COX-2, a pivotal player for UV-induced skin cancer promotion^21–23^, is involved in VRCZ-induced skin cancer formation. Generally, COX-2-PGE_2_ pathway is thought to mediate the promotion and progression phases of UV-induced skin cancers, as animal models and clinical studies using COX-2 inhibitor have shown the role of COX-2-PGE_2_ pathway^24^. We treated HaCaT KCs with VRCZ (1–100 µM) and DMSO (control) for 2 h. VRCZ elevated *COX-2* mRNA dose-dependently as assessed by real-time-PCR (RT-PCR) (Fig. 3A). VRCZ upregulation of *COX-2* was also seen in NHEK and cSCC cell line (Fig. 3B). HaCaT cells were positively stained for COX-2, when they were treated with VRCZ at 30 or 100 µM for 6 hours (Fig. 3C). In LSE, IHS also exhibited increment of COX-2 expression by 30 µM VRCZ in the epidermis, especially in the perinuclear area of the basal cells, compared with the vehicle control (Fig. 3D). This increase was confirmed at mRNA level (Fig. 3E). Furthermore, we found that PGE_2_, an enzymatic product of COX-2, was increased in HaCaT cells by VRCZ in a dose dependent manner (Fig. 3F).

**Figure 4:**
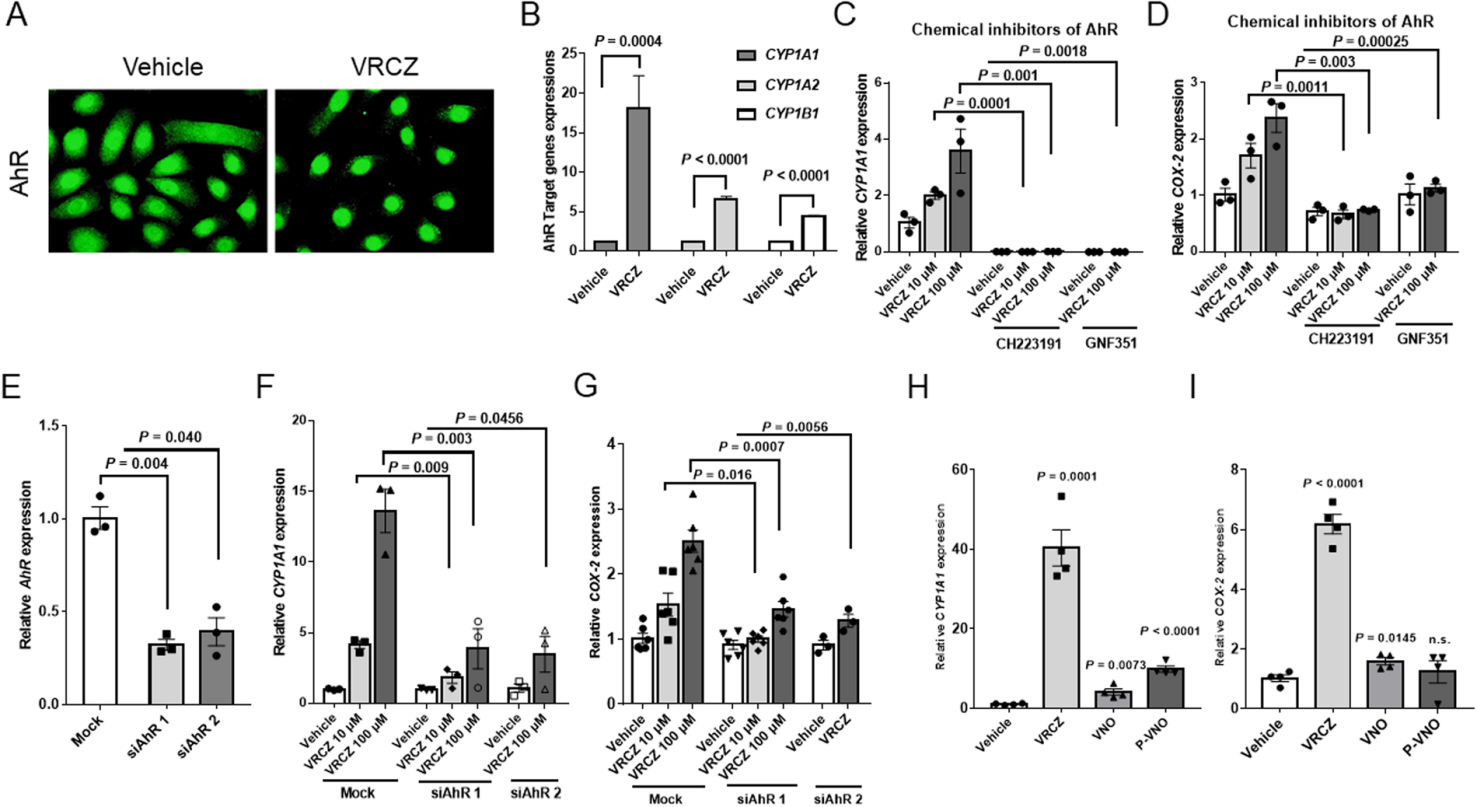
Induction of AhR signaling and COX-2 expression by VRCZ. (**A**) HaCaT cells were exposed to 30 μM VRCZ or vehicle (DMSO; 0.07%) for 3 hours. Cells were then fixed and stained with the polyclonal IgG rabbit anti-human AhR Ab followed by incubation with FITC-labeled anti-rabbit IgG secondary antibody. VRCZ induced the translocation of AhR to nucleus. Scale bar: 50 µm. Representative date (*n* = 3). (**B**) HaCaT KCs were exposed to 100 μM VRCZ or vehicle for 2 hours. mRNA levels of AhR target genes (*CYP1A1*, *1A2* and *1B1*) were analyzed using real time RT-PCR. Bars indicate mean ± SEM (*n* = 3). Student’s *t* test. (**C**,**D**) HaCaT cells were preincubated with AhR antagonist CH223191 (10 µM) or GNF351 (500 nM) for 30 minutes and incubated with various concentrations of VRCZ at 10 or 100 μM for 2 hours. The expression levels of AhR-target gene *CYP1A1* and *COX-2* were monitored. The results are expressed as folds of induction after VRCZ treatments. Bars indicate mean ± SEM (*n* = 3). Student’s *t* test. (**E–G**) HaCaT cells transfected with siRNA-control or siRNA-AhRs were exposed to VRCZ or vehicle for 2 hours. The mRNA expressions of *AhR*, *CYP1A1* and *COX-2* were analyzed by real-time RT-PCR. Bars indicate mean ± SEM (E, F: *n* = 3; G: *n* = 6). Student’s *t* test. (**H**,**I**) Relative *CYP1A1* and *COX-2* mRNA expression augmented by VRCZ and its metabolites (VNO and P-VNO) were examined in HaCaT cells after 2 hours incubation with each substance. Date are normalized to GAPDH and expressed as fold increase from DMSO-treated controls. Bars indicate mean ± SEM (*n* = 3). Student’s *t* test.

To address the mechanism of the upregulation of COX-2 by VRCZ, we focused on AhR, because KCZ, another azole agent, is an activator of AhR^25^, and 2,3,7,8-tetrachlorodibenzp-p dioxin (TCDD), a typical AhR ligand, or other AhR agonists increases COX-2 expression^16,26,27^. HaCaT cells were treated with VRCZ (30 µM) or DMSO as control, and AhR nuclear translocation was monitored. VRCZ, but not DMSO alone, caused translocation of AhR to the nuclei within 4 hours (Fig. 4A), indicating an operation of AhR-mediated signaling by VRCZ. Transcription of AhR-targeting genes, including *CYP1A1*, *1A2* and *1B1*, were also elevated as assessed by RT-PCR (Fig. 4B). Thus, VRCZ is an activator of AhR in human KCs.

To further investigate the involvement of AhR signaling pathway in VRCZ-mediated COX-2 expression, we first examined the effect of an AhR antagonists, CH223191^28^ and GNF351 on the expression levels of *CYP1A1* and *COX-2*. Both chemical inhibitors completely inhibited the VRCZ-induced transcription of *CYP1A1* (Fig. 4C) and *COX-2* (Fig. 4D). We also monitored the VRCZ response after small interference AhRs (siAhR 1 and 2)-mediated knockdown of AhR in HaCaT cells. This interferences reduced the AhR expression by 70% (Fig. 4E). The siAhRs significantly downregulated the mRNA expression of its classical target gene *CYP1A1* (Fig. 4F) and the expression of *COX-2* (Fig. 4G). Thus, VRCZ induces COX-2 expression *via* AhR signaling pathway. In contrast to VRCZ, VRCZ metabolites exerted only slight effects on the AhR target genes, *CYP1A1* (Fig. 4H) and *COX-2* (Fig. 4I).

### VRCZ upregulates COX-2 expression in an AhR-ARNT dependent and is independent of AhR-Nrf2-Nqo-1 signaling pathway

**Figure 5:**
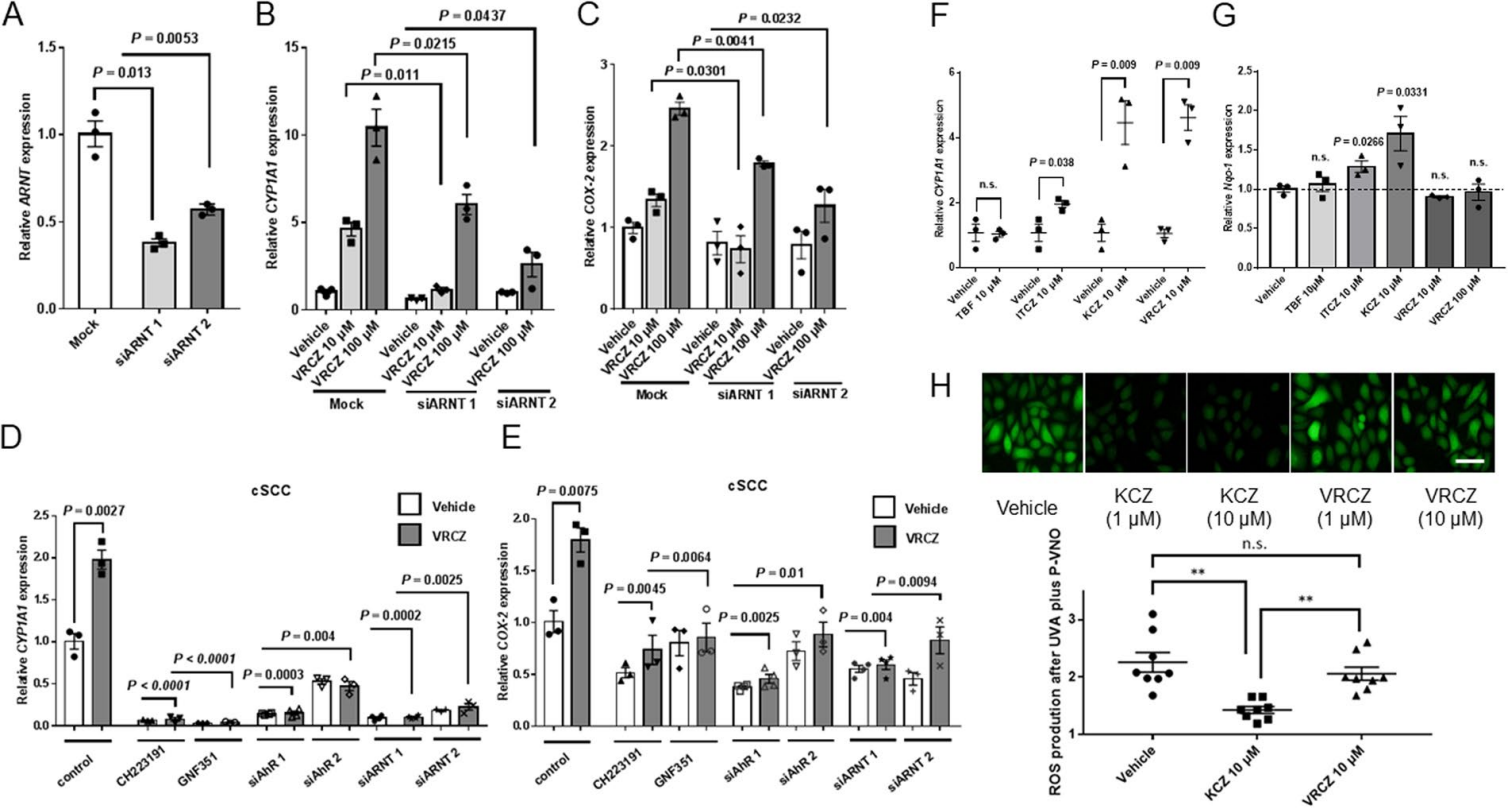
VRCZ upregulates COX-2 expression in an AhR-ARNT dependent and is independent of AhR-Nrf2-Nqo-1 signaling pathway. To elucidate whether the VRCZ-induced expression of COX-2 is classical genomic (ARNT dependent) or non-genomic, we used siARNTs. HaCaT cells transfected with siRNA-control or siRNA-ARNT 1 or 2 (**A**) were exposed to VRCZ or vehicle (0.07% DMSO) for 2 hours (**B**,**C**). The mRNA expressions of *ARNT*, *AhR*, *CYP1A1* and *COX-2* were analyzed by real-time RT-PCR. Bars indicate mean ± SEM (*n* = 3). Student’s *t* test. (**D**,**E**) To further confirming the results, we also using the human cutaneous SCC cell line HSC for the inhibitory experiments. (**F**,**G**) To identify the differences between VRCZ and other anti-fungals, terbinafine hydrochloride (TBF), itraconazole (ITCZ) and ketoconazole (KCZ), HaCaT KCs were exposed to 10 or 100 μM VRCZ or the 10 μM anti-fungal agents (TBF, ITCZ and KCZ) for 2 hours. mRNA levels of AhR target gene *CYP1A1* and antioxidant enzyme *Nqo-1* were analyzed by real time RT-PCR. Bars indicate mean ± SEM (*n* = 3). Student’s *t* test. (**H**) The effects of KCZ or VRCZ on ROS production by P-VNO plus UVA in NHEKs. After treatment with DMSO (0.07%), KCZ (1 μM or 10 μM) and VRCZ (1 μM or 10 μM) for 24 hours, NHEKs were exposed to P-VNO (30 μM) for 30 minutes. Then, they were irradiated with 2.0 J/cm^2^ (1.2 mW/sec for 30 min) UVA. Immediately after irradiation, NHEKs were subjected to ROS experiments. Panels is representative image from 2 replicate experiments and data are presented as means ± SEM (*n* *=* 8). Scale bar: 50 µm.

Several studies have demonstrated that an AhR dependent, but not classical (genomic), pathway operates in the dioxin-inducing COX-2 elevation. This non-genomic pathway is conducted by the calcium ion influx as the first event^29^. Non-genomic pathway is characterized by the lack of participation of ARNT, which interacts with the AhR-ligand in the nucleus and forms the AhR-ligand-ARNT complex^26,29^. To elucidate whether the VRCZ-induced expression of COX-2 is genomic or non-genomic, we therefore used siARNTs (siARNT 1 and 2). The siARNTs significantly inhibited the expression of *ARNT* (Fig. 5A). They also downregulated *CYP1A1* expression (Fig. 5B) and *COX-2* expression (Fig. 5C), indicating that VRCZ upregulates COX-2 in an AhR-ARNT dependent manner, namely *via* the classical pathway. We also obtained similar actions of the AhR inhibitors in human cutaneous SCC cell line HSC, further confirming our findings (Fig. 5D,E).

Azole antifungal ketoconazole (KCZ) is identified as an activator of AhR^25^. We compared the ability of VRCZ to increase *CY1A1* expressions with those of KCZ and other two anti-fungals, terbinafine hydrochloride (TBF) and itraconazole (ITCZ). We observed that KCZ also increased *CY1A1* mRNA levels similarly to VRCZ, while neither TBF nor ITCZ were stimulatory (Fig. 5F).

The capacity of VRCZ to induce skin cancers is clinically higher than that of KCZ. We hypothesized that the difference exists between these two agents and tested their abilities, focusing on nuclear factor erythroid 2-related factor-2 (Nrf2) signaling. KCZ can activate the Nrf2 *via* AhR signaling and eliminate ROS, resulting in protection of DNA damage^25^. This redox reaction represents the anti-inflammatory effect of KCZ^25^. We measured mRNA expression of antioxidant enzyme NAD(P)H:quinone oxidoreductase 1 (*Nqo-1*), which reflects Nrf-2 signaling activation^30^. KCZ activated the Nrf2-Nqo-1 system upon AhR stimulation (Fig. 5G), as reported previously^25,30^, meanwhile, VRCZ did not. Next, we compared the effects on redox between KCZ and VRCZ. The inhibitory effects of these antifungals on ROS were examine in stimulation with tumor necrosis factor-α (TNF-α) (Supplemental Fig. 2) or UVA plus P-VNO (Fig. 5H). The results demonstrated the difference between KCZ and VRCZ, as KCZ has a cell protective action, while VRCZ *per se* is unable to eliminate ROS produced by P-VNO plus UVA. Namely, COX-2 upregulation *via* AhR signaling seems to be the common pathway for azole agents, AhR-Nrf-2-Nqo-1 signaling may operates with certain azoles such as KCZ, but not VRCZ.

## Discussion

**Figure 6:**
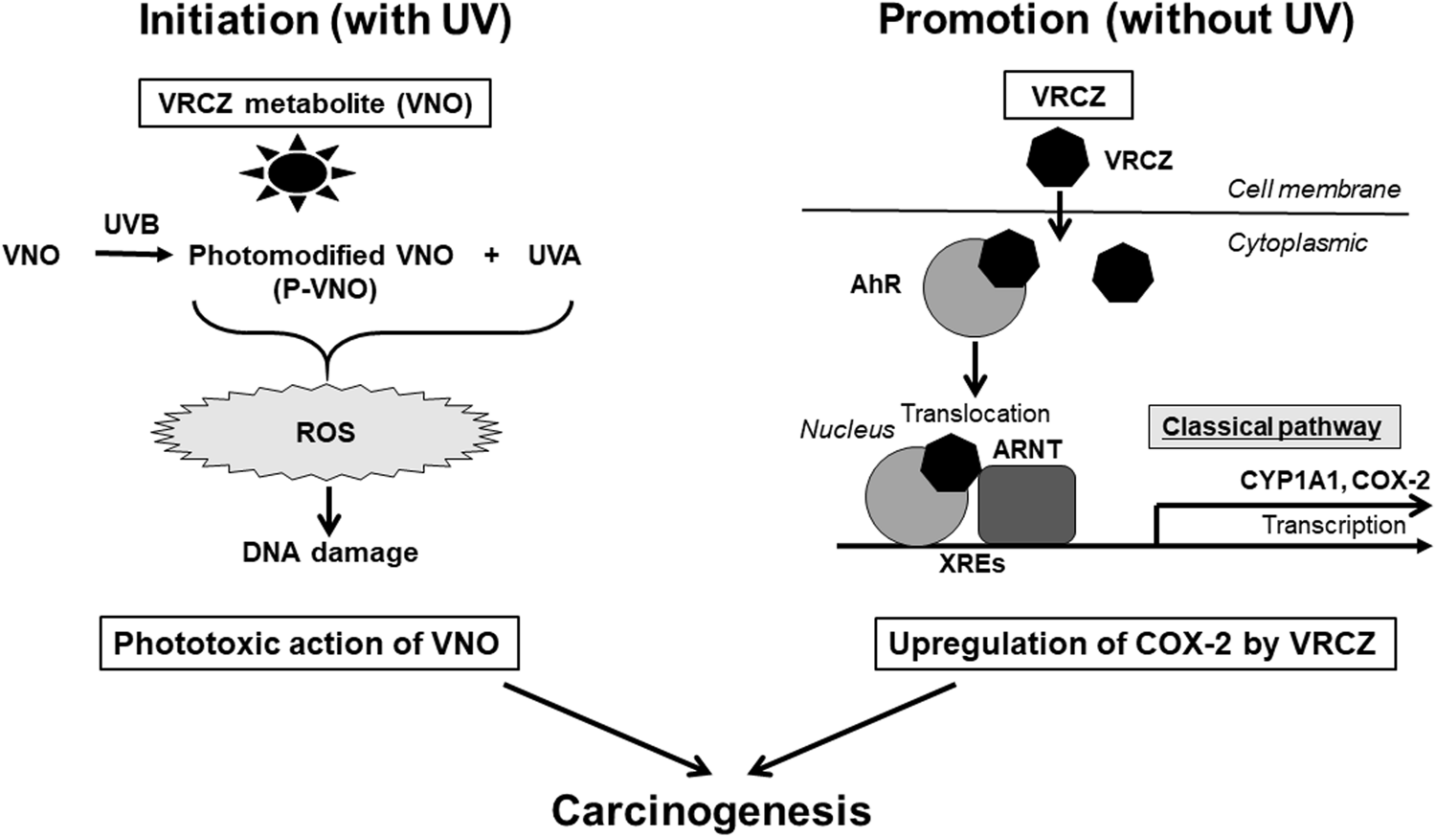
Schematic summary of mechanisms underlying VRCZ-induced skin cancer. VRCZ metabolites plus UVA generated reactive oxygen species and resultant DNA damage of the epidermis, but did not induce substantial apoptosis in human keratinocytes (KCs). VRCZ *per se* stimulates aryl hydrocarbon receptor (AhR) and upregulates COX-2 in an AhR-ARNT dependent manner of the classical (genomic) pathway. AhR, aryl hydrocarbon receptor; ARNT, AhR nuclear translocator; CYP, cytochrome P450; P-VNO, photo-modified-VNO; VNO, Voriconazole *N*-oxide; VRCZ, voriconazole; XRE, xenobiotic responsive element.

VRCZ has an absolutely unique action for skin cancer development. In this study, we demonstrated that the VRCZ metabolites are phototoxic agents serving as an initiator of tumorigenesis, and VRCZ *per se* is an AhR-COX-2 activator functioning as a promoter of tumor development (Fig. 6). In the initiation phase, the lack of apoptosis induction in P-VNO-phototreated KCs may allow oxidative stress-damaged tumor cells to survive. More importantly, VRCZ primarily promotes the tumor development of UV-damaged KCs by P-VNO plus UVA in an AhR dependent fashion by increasing COX-2 expression. Thus, the unique carcinogenetic potential of VRCZ is considered to stem from different action points encompassing both the initiation and promotion phases.

In the initiation phase, we focused on the VRCZ metabolites, especially its photo-modified agent, P-VNO. It was reported that the serum concentration of VNO was 6.10 (1.83) µM at C_max_ (SD)^31^ or 10 µM^32^ at most, when patients were administered with the usual dose of VRCZ. Although VNO concentration in the epidermis is unknown, epidermal keratinocytes seem to be exposed to the comparable concentration of VNO. The expression of several cytochrome p450 enzymes in the skin which metabolize VRCZ^2,5,33^ is also supportive for this notion. We found that P-VNO plus UVA induced ROS production strongly at 100 µM and moderately at 10 µM after 2.0 J/cm^2^ UVA, suggesting that ROS is yielded in the clinical settings as well as the cultured KCs. A previous report showed that P-VNO absorbs UVA, leading to ROS generation and DNA damage^14^. We further examined the qualitative analysis of P-VNO by HPLC and found that UVB exposure of VNO definitely generated a photo-modified substance, P-VNO. Our finding with the LSE model together with observations by other group^14^ consistently shows that P-VNO plus UVA causes DNA damage. However, we found that the capacity of P-VNO with UVA to induce apoptosis is markedly low as compared with the other known photosensitizing drugs. In general, a non-toxic level of ROS attenuates or modifies cell functions and structures in KCs and enhances a pro-survival pathway, although excess ROS damages KCs to cell death^21,34^. Our results suggest that the low ROS production by VRCZ metabolite plus UVA results in paucity of apoptosis and allows DNA-damaged cells to survive. It is tempting to speculate that patients can be given VRCZ even for a long period because of the low ROS-producing phototoxic property of P-VNO. Since the development of VRCZ-skin cancer requires not only UVB but also UVA, the mutation spectrum is complex or might be influenced by another bystander molecule.

Only the above initiation-related VRCZ features in the context of UV may be insufficient to explain the clinically extensive and aggressive characteristics of cSCC. AhR is a ligand-dependent transcription factor with basic-helix-loop-helix/PER-ARNT-SIM homology region family^35^ that mediates a wide range of biological and toxicological effects upon exposure to a structurally diverse variety of synthetic and naturally occurring chemicals^36^. The best-characterized high-affinity ligands for AhR include a variety of ubiquitous hydrophobic environmental contaminants such as the halogenated or non-halogenated aromatic hydrocarbons. Intriguingly, azole antifungal agent KCZ is also identified as an AhR activator in human hepatocytes^15^ and KCs^25^. When environmental pollutants, such as TCDD and 3-methylcholanthrene, bind to AhR, the ligand-activated AhR translocates to the nucleus and binds to its heterodimerization partner ARNT^37^. The heterodimer AhR/ARNT binds to xenobiotic responsive elements (XREs), which are enhancer DNA elements located in the 5′-flanking region of the target genes^38^. As expected, VRCZ treatment induced translocation of AhR to nucleus and upregulate AhR target genes, *CYP1A1*, *CYP1A2* and *CYP1B1* in KCs, indicating that VRCZ serves as AhR activator. Even non-dioxin chemicals bind to AhR by virtue of specific amino acids within AhR ligand-binding domains^39^. Similar to KCZ^15^, VRCZ is considered to activate AhR signaling in KCs.

We investigated VRCZ/AhR/COX-2 signaling pathway to elucidate the mechanism of VRCZ carcinogenesis. It has been reported that increased levels of COX-2 are associated with the etiology of a variety of tumors, including skin cancers^40,41^. Oncogenes, growth factors, cytokines, chemotherapy and tumor promoters stimulate COX-2 transcription *via* protein kinase C- and RAS-mediated signaling^42,43^. Furthermore, in cSCC accelerated by BRAF inhibitors, COX-2 inhibitor functions as a chemopreventive agent^44^. Intriguingly, several lines of evidence have suggested that COX-2 is induced in an AhR dependent manner. AhR ligand dioxin increases COX-2 in different cell types^26,27,45^. Furthermore, endogenous AhR ligand 6-formylindolo[3,2-*b*]carbazole (FICZ) induces AhR-mediated COX-2 in human KCs^46^. In our study, VRCZ upregulated COX-2 in a dose-dependent manner, and its upregulation was canceled by pretreatment of KCs with an AhR antagonist or siAhR, confirming the AhR dependency of VRCZ-induced COX-2 upmodulation.

Previous studies documented that the TCDD-induced COX-2 expression is mediated by the non-genomic pathway in rats^27^ and mice^45^. Meanwhile, another study using breast cancer cells demonstrated TCDD-induced binding of the AhR to the COX-2 promoter, suggesting that the coordinated recruitment of the AhR, p300, and histone H4 acetylation may lead to the activation of COX-2 expression through the classical (genomic) pathway^16^. In our study, siRNA-mediated knockdown of ARNT downregulated both *CYP1A1* and *COX-2*, indicating that VRCZ upregulates COX-2 in an AhR-ARNT dependent manner of the classical pathway.

COX-2 contributes to carcinogenesis with PGE_2_, which exerts its autocrine/paracrine effects on target cells by coupling to G-protein-coupled receptors (E-series prostanoid receptors) and mediates tumor progression and/or angiogenesis^47,48^. In inflammation, PGE_2_ has two aspects of pro- and anti-inflammatory effects. Sertaconazole activates the p38/COX-2/PGE_2_ signaling and has an anti-inflammatory effect both *in vitro* and *in vivo*^49^. VRCZ-induced PGE_2_ production may cause an anti-inflammatory effect on the formation of AK. Thus, VRCZ/AhR/COX-2/PGE_2_ axis promotes the skin tumor progression. In addition, the inhibition of immune cell infiltration by VRCZ at least partly allows the tumor cells to escape from the antitumor immunity. We believe that the VRCZ-induced AKs showing highly acanthotic and scarcely inflammatory histolopathology support our speculation.

In conclusion, our findings suggest that VRCZ primarily promotes the tumor development of DNA-damaged KCs, those are caused by the phototoxic moieties of VRCZ metabolites which may participate in the initiation phase. Therefore, during VRCZ therapy, sun exposure protection is essential to prevent photo-carcinogenesis caused by VRCZ metabolites plus UV. Chemoprevention with selective COX-2 inhibitors may be helpful to depress the development of skin cancers derived from DNA-damaged KCs.

## Methods

### Patients and clinical specimens

Skin tumor samples and patients’ clinical information were collected from the patients treated between 2012 and 2016 at the Hamamatsu University School of Medicine. H&E-stained sections were scanned with a digital image scanner (NanoZoomer 2.0-HT; Hamamatsu Photonics, Hamamatsu, Japan) and analyzed with its software. For microscopic quantification of epidermal thickness (a distance between the bottom of the basal layer and the granular layer), 4-time measurements were performed at an interval of 500 µm for each sample, and the mean of them represented the value of individual cases. The number of infiltrating cells was counted in five fields of each 0.196 mm^2^. The use of clinical and histopathological study of patients was approved by the ethical committee of Hamamatsu University School of Medicine (approval E14-2701). Written informed consent was obtained from the patients upon skin biopsy. The study was conducted in full concordance with the principles of the declaration of Helsinki.

### Reagents

VRCZ and VNO were purchased from Toronto Research Chemicals Inc. (North York, ON, Canada). They were dissolved in DMSO as 150 mM stock solutions and were used immediately or stored at −20 °C until used. HPLC-grade acetonitrile and methanol were purchased from Wako Pure Chemicals (Osaka, Japan). Photoproduct of VNO (P-VNO) was generated from VNO by irradiating with UVB irradiation (500 mJ/cm^2^). Afloqualone (AQ) was obtained from Tanabe Seiyaku Co., Osaka, Japan. The solution of PBS contained 0.3 mM AQ was prepared as described previously^20^. Sparfloxacin, the AhR antagonists CH223191 and GNF351 were procured from Sigma-Aldrich, St. Louis, MO. KCZ, TBF and ITCZ were purchased from Wako Pure Chemicals, Osaka, Japan. Recombinant human TNF-α was purchased from R&D Systems, Minneapolis, MN.

### UV sources

Black light (FL20SBLB) emitting UVA ranging from 320 to 400 nm with a peak emission at 365 nm, and sunlamp (FL20SE) emitting UVB ranging from 280 to 320 nm with a peak emission at 305 nm were purchased from Toshiba Electric Co., Tokyo, Japan. With a Dermaray UV meter, Terumo Clinical Supply Co., Gifu, Japan, the energy output of three 20-W tubes of black light at distance of 20 cm was 2.2 mW/cm^2^ at 365 nm and 0.17 mW/cm^2^ at 305 nm, and that of three sunlamps was 1.2 mW/cm^2^ at 305 nm and less than 0.01 mW/cm^2^ at 365 nm. In the UVA irradiation experiments, the cells or human living skin equivalent (LSE) were placed in plastic dishes and irradiated with three tubes of black light at a distance of 20 cm through a pane of 3 mm thick glass to ensure that no radiation below 320 nm reached the solutions^20^. Under these conditions, the irradidance that reached to the solutions was 1.2 W/cm^2^ at 365 nm.

### Spectrophotometry

Spectroscopic studies were conducted at room temperature on solutions in 96-well plates. Absorption spectra were recorded on Synergy HT (Bio-Tek, Winooski, VT) under the following settings-scan range 230 to 400 nm; slit width, 1.0 nm; scan rate of 5 nm/s.

### HPLC analysis

VNO and P-VNO were analyzed using an HPLC system (Shimadzu Corp, Kyoto, Japan) consisted of a DRU-20A_3_ degasser, LC-20AD pump, SIL-20ACHT auto injector, CTO-20A column heater, and SPD-M20A diode array detector. Data were collected and analyzed by LabSolutions software (version 5.73). The separations were conducted as described previously^50^. The wavelength of the photodiode array UV detector was set at 340 nm. The injection volume was 50 µL.

### Cell culture studies

Normal human epidermal keratinocytes (NHEKs) and human fetal fibroblasts were purchased from Kurabo Industries, Osaka, Japan. They were grown in serum-free keratinocyte growth medium Epilife (Invitrogen, Carlsbad, CA), containing 0.06 mM Ca^2+^ and 1 × Epilife Defined Growth Supplement (EDGS; Invitrogen) at 37 °C under standard tissue culture conditions. They were used at third passage in LSE experiments. HaCaT cells (obtained from Department of Dermatology, University of Occupational and Environmental Health, in 2011, when *Mycoplasma* infection was tested)^51^ were cultured in DMEM (Gibco BRL Life Technology, Gaithersburg, MD) supplemented with 10% heat-inactivated FCS, 100 units/ml penicillin, and 100 µg/ml streptomycin, 1% _L_-glutamine (all from Gibco BRL) in a humidified atmosphere of 5% CO_2_ and air at 37 °C. The human cutaneous SCC cell line HSC1 was purchased from the Japan Health Sciences Foundation (cell bank registration JCRB1015, in 2015, when *Mycoplasma* infection was tested) and cultured in DMEM with 20% FCS. Growth supplement was omitted 24 h before experiments.

### ROS measurement

To quantify the magnitude of ROS formation by agents plus UVA in live cells, we used OxiSelect^TM^ Intracellular ROS Assay Kit (Cell Biolabs, San Diego, CA) according to the manufacturer’s directions. HaCaT or NHEK cells in 96-well cell culture plates were incubated with 2′,7′-dichlorofluorescein diacetate (DCFH-DA) (100 µM) for 30 min at 37 °C. DCFH-DA-loaded cells were treated with agents for 30 min, and irradiated with varying doses of UVA. In the ROS inhibitory study, cells were pretreated with KCZ or VRCZ (1 or 10 µM) for 24 hours. Then stimulate them with TNF-α (10 ng/ml) or P-VNO plus UVA. Green fluorescence (excitation: 495 nm; emission: 515 nm) was detected by a plate reader, Synergy HT (Bio-Tek). Immediately after irradiation, the cells were subjected to experimental treatments.

### Human living skin equivalent (LSE) model

The method used to prepare LSE was described previously^52^. Briefly, a collagen gel (the mixture of porcine collagen solution, DMEM, FCS and 0.1 N NaOH) was added to each culture insert (Transwell-COL, membrane pore size 3 µm; Costar, Corning, NY) in a 6-well Costar culture plate (Corning). Following polymerization of the gel in the inserts, a solution of fibroblast-containing collagen was applied to each insert. When the fibroblast-containing gel was polymerized, DMEM supplemented with 10% FCS and ascorbic acid (final concentration 50 ng/mL) was added. The culture medium was changed twice per week. The gel was used to construct the LSEs. Five days after the dermal component was prepared, keratinocytes in MCDB 153 type II were seeded onto the concave surface of the contracted gel. They were kept submerged in culture medium for 2 days. When the keratinocytes reached confluence, the LSE was lifted to an air-liquid interface and cornification medium was added. The medium was changed every other day. Two weeks after airlift, the LSEs were used for experiments. The LSE samples were embedded in paraffin for immunohistochemical staining (IHS) or frozen in liquid nitrogen, and fresh frozen tissue was stored at −80 °C for quantitative real-time polymerase chain reaction analysis and IHS. We performed at least three independent studies, which gave similar results. A representative experiment is shown in the figures.

### Immunostaining of monolayer KCs and LSE

Direct immunofluorescence labeling was performed with antibodies to AhR (1:200) (sc-5579, Santa Cruz Biotechnology, CA), 8-hydroxy-2′-deoxyguanosine (8-OHdG) (1:1000) (ab2623, Abcam, Tokyo, Japan) and COX-2 (1:100) (ab15191, Abcam, Tokyo, Japan) under a microscope equipped with Plan APOCHROMAT (Olympus). Alexa Fluor 488 or 594 (Molecular Probes, Inc., Eugene, OR) were used as secondary antibodies. DAPI (Molecular Probes, Inc.) was used to visualize nuclei. For indirect immunostaining of LSEs for COX-2 (1:200; Abcam), deparaffinized specimens were autoclaved in 10 mM citrate buffer (pH 6.0) for 10 minutes at 120 °C to retrieve the antigenic epitopes and then subjected to COX-2 expression analysis by using the avidin-biotin complex method.

### Apoptosis analysis using flow cytometry

HaCaT cells in 24-well cell culture plate were incubated with agents for 30 minutes, and then irradiated with varying doses of UVA. They were further cultured for 8 hours (24 and 48 hours in the P-VNO plus UVA experiment) and harvested by trypsinization. They were washed with cold PBS and stained with Annexin V-FITC apoptosis detection kit I (BD Biosciences, Pharmingen, San Diego, CA) according to the manufacturer’s instructions. Cells positive for 7-amino-actinomycin D (7-AAD), Annexin V-FITC or both were quantified by flow cytometry using a Becton Dickinson FACSCanto II (BD Biosciences, San Jose, CA). Results were analyzed with FlowJo software (TreeStar, Ashland, OR).

### Quantitative real-time PCR analysis

RNA was extracted from the samples using the RNeasy Mini Kit (Qiagen, Valencia, CA) according to the manufacturer’s protocol. cDNA was reverse transcribed from total RNA using the TaqMan RT reagents (Applied Biosystems, Foster City, CA). mRNA expression was analyzed with SYBR®GreenERTM qPCR Reagent system (Invitrogen) using the ABI PRISM 7000 sequence detection system (Applied Biosystems). The primers were as follows: AhR: forward 5′-ATCACCTACGCCAGTCGCAAG-3′ and reverse: 5′-AGGCTAGCCAAACGGTCCAAC-3′′;

ARNT: forward 5′-CCCTAGTCTCACCAATCGTGGATC-3′ and reverse: 5′- GTAGCTGTTGCTCTGATCTCCCAG-3′; COX-2: forward 5′-GAATGGGGTGATGAGCAGTT-3′ and reverse: 5′-CAGAAGGGCAGGATACAGC-3′; CYP1A1: forward 5′- TCACAGACAGCCTGATTGAG -3′ and reverse: 5′- GATGGGTTGACCCATAGCTT -3′; CYP1A2: forward 5′-GGGCACTTCGACCCTTACAA-3′ and reverse: 5′-GCACATGGCACCAATGACG-3′; CYP1B1: forward 5′-TGCCTGTCACTATTCCTCATGCCA-3′ and reverse: 5′-ATCAAAGTTCTCCGGGTTAGGCCA-3′; Nqo1: forward 5′-GGATTG GACCGAGCTGGAA-3′ and reverse: 5′-AATTGCAGTGAAGATGAA GGCAAC-3′; and GAPDH: forward 5′-ACCCACTCCTCCACCTTTGA-3′ and reverse: 5′- CTGTTGCTGTAGCCAAATTCGT-3′. GADPH was used as the housekeeping genes. The relative expression was calculated according to the comparative threshold cycle method (2–ΔΔCt).

### Measurement of PGE_2_ release

HaCaT cells (5 × 10^4^ cells/well) were seeded in 24-well plates in 0.4 ml DMEM, and allowed to adhere overnight. The cells were washed and incubated with FCS-free DMEM for 24 hours. The culture was incubated in the presence of vehicle (0.07% DMSO) or indicated concentrations of VRCZ for 6 hours. The supernatant PGE_2_ amounts were quantified using a PGE_2_ ELISA kit (highly sensitive kit for inflammation and eicosanoid research, ADI-900-001; Enzo Life Sciences, Farmingdale, NY) according to the manufacturer’s protocol.

### Small interfering RNA (siRNA)-targeted gene silencing

SiRNA targeted against AhR 1 and 2 (siRNA identification numbers s1200 and s1198) and AhR nuclear translocator (ARNT 1 and 2) (s1613 and s1615) were purchased from Ambion (Austin, TX) and were used to interfere with human AhR and ARNT expression in NHEKs, HaCaT KCs and the human cutaneous SCC cell line HSC1. Transfection was performed by using HiPerFect Transfection kit (Qiagen, Courtaboeuf, France), and an equivalent amount of scrambled siRNA was employed as negative control. Cells in 24-well plates were incubated with 0.5 ml culture medium containing 10 nM siRNA and 3.0 µl of HiPerFect reagent. After 48 hours cultivation, siRNA-transfected cells were starved for 24 hours, and treated with VRCZ for 2 hours. Cells were harvested for transcriptional analysis as described above.

### Statistical analysis

Data were given as mean ± SEM of at least 3 biological replicates. Means of 2 groups were compared using a 2-tailed Student’s *t* test. Multiple comparisons between groups were performed using 1-way ANOVA with Tukey-Kramer multiple comparison tests. Difference with *P* < 0.05 was considered significant. Statistical analysis was performed with Graph Pad Prism version 7.00 for Windows (Graph Pad software, San Diego, CA).

## Electronic supplementary material

Supplementary Information 
