# Supplementary material for: Voriconazole-induced photocarcinogenesis is promoted by aryl hydrocarbon receptor-dependent COX-2 upregulation
Source: Sci Rep. 2018 Mar 22;8:5050. doi: 10.1038/s41598-018-23439-7 (PMC5864729; doi:10.1038/s41598-018-23439-7)
Supplement: Supplementary file 1 — Supplementary Information [file 41598_2018_23439_MOESM1_ESM.pdf]

Voriconazole-induced photocarcinogenesis is promoted by aryl hydrocarbon receptor-dependent COX-2 upregulation

Shigeki Ikeya[1*], Jun-ichi Sakabe[1], Takahiro Yamada[2], Takafumi Naito[2], and Yoshiki Tokura[1]

[1]Department of Dermatology, Hamamatsu University School of Medicine, 1-20-1 Handayama, Higashi-ku, Hamamatsu 431-3192, Japan.
[2]Department of Hospital Pharmacy, Hamamatsu University School of Medicine

Supplemental figure 1

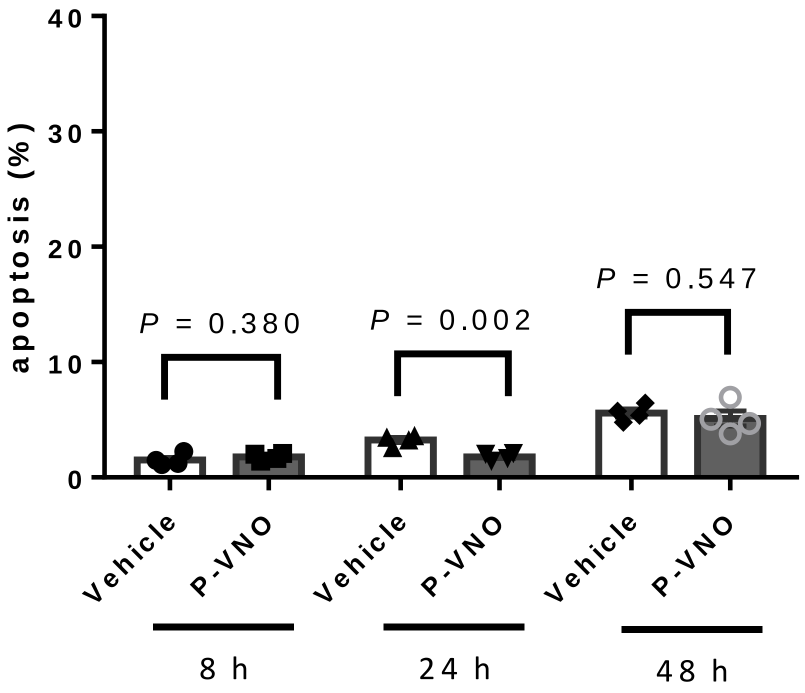

Supplemental figure 1. Time course of the apoptosis induced by P-VNO plus UVA.
HaCaT KCs were pretreated P-VNO (100 μM) or vehicle and irradiated with UVA at 2.0 J/cm^2 (1.2 mW/sec for 30 min) at different time points (8, 24 and 48 hours after UVA irradiation). Apoptosis was analyzed by flow cytometry. The results were expressed as the mean percentage ± SEM of apoptotic cells (n = 4). **P < 0.01. Student's t test.

Supplemental figure 2

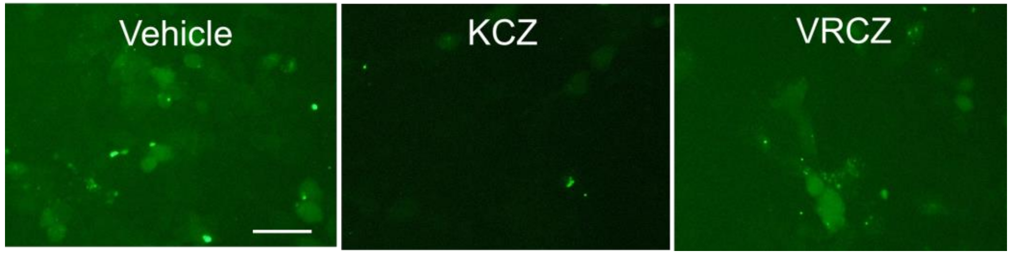

Supplemental figure 2. Effect of antifungals on ROS by the tumor necrosis factor-α (TNF-α). Ketoconazole inhibits ROS production by the tumor necrosis factor-α (TNF-α), meanwhile, VRCZ does not. HaCaTs were pretreated with DMSO (0.07%), KCZ or VRCZ (10 μM) for 24 hours. Then stimulate them with TNF-α (10ng/ml). The magnitude of ROS formation was evaluated with DCFH-DA (Green fluorescence). Panels is representative image from 2 replicate experiments.
